# Preferences for Mobile App Features to Support People Living With Chronic Heart Diseases: Discrete Choice Study

**DOI:** 10.2196/58556

**Published:** 2025-04-25

**Authors:** Sumudu Avanthi Hewage, Sameera Senanayake, David Brain, Michelle J Allen, Steven M McPhail, William Parsonage, Tomos Walters, Sanjeewa Kularatna

**Affiliations:** 1 Australian Centre for Health Services Innovation and Centre for Healthcare Transformation, School of Public Health and Social Work, Queensland University of Technology, 61 Musk Avenue, Brisbane, 4059, Australia, +61 07 3388 6077; 2 Health services and systems research, Duke-NUS Medical School, 8, College Road, Singapore; 3 National Heart Research Institute Singapore, National Heart Centre, 5, Hospital Drive, Singapore; 4 Digital Health and Informatics Directorate, Metro South Health, Queensland, Australia; 5 Department of Cardiology, Royal Brisbane and Women's Hospital, Queensland, Australia; 6 Queensland Cardiovascular Group, Queensland, Australia; 7 Faculty of Medicine, The University of Queensland, Queensland, Australia

**Keywords:** digital health technologies, user preferences, latent class model, monitoring vital signs, adoption rates, app, chronic heart disease, heart disease, digital health, effectiveness, user, mobile health app, self-navigate, health education, symptom, monitoring, adoption

## Abstract

**Background:**

Using digital health technologies to aid individuals in managing chronic diseases offers a promising solution to overcome health service barriers such as access and affordability. However, their effectiveness depends on adoption and sustained use, influenced by user preferences.

**Objectives:**

This study quantifies the preferences of individuals with chronic heart disease (CHD) for features of a mobile health app to self-navigate their disease condition.

**Methods:**

We conducted an unlabeled web-based choice survey among adults older than 18 years with CHD living in Australia, recruited via a web-based survey platform. Four app attributes—ease of navigation, monitoring of blood pressure and heart rhythm, health education, and symptom diary maintenance—were systematically chosen through a multistage process. This process involved a literature review, stakeholder interviews, and expert panel discussions. Participants chose a preferred mobile app out of 3 alternatives: app A, app B, or neither. A D-optimal design was developed using Ngene software, informed by Bayesian priors derived from pilot survey data. Latent class model analysis was conducted using Nlogit software (Econometric Software, Inc). We also estimated attribute importance and anticipated adoption rates for 3 app versions.

**Results:**

Our sample included 302 participants with a mean age of 50.5 (SD 18.2) years. Latent class model identified 2 classes. Older respondents with education beyond high school, prior experience with mobile health apps, and a positive perception of app usefulness were more likely to be in class 1 (257/303, 85%) than in class 2 (45/303, 15%). Class 1 membership preferred adopting a mobile app (app A: β coefficient 0.74, 95% uncertainty interval (UI) 0.41-1.06; app B: β coefficient 0.53, 95% UI 0.22-0.85). Participants favored apps providing postmonitoring recommendations (β coefficient 1.45, 95% UI 1.26-1.64), tailored health education (β coefficient 0.50, 95% UI 0.36-0.64), and unrestricted symptom diary entry (β coefficient 0.58, 95% UI 0.41-0.76). Class 2 showed no preference for app adoption (app A: β coefficient −1.18, 95% UI −2.36 to 0.006; app B: β coefficient −0.78, 95% UI −1.99 to 0.42) or any specific attribute levels. Vital sign monitoring was the most influential attribute among the 4. Scenario analysis revealed an 84% probability of app adoption with basic features, rising to 92% when app features aligned with respondents’ preferences.

**Conclusions:**

The study’s findings suggest that designing preference-informed mobile health apps could significantly enhance adoption rates and engagement among individuals with CHD, potentially leading to improved clinical outcomes. Adoption rates were notably higher when app attributes included easy navigation, vital sign monitoring, feedback provision, personalized health education, and flexible data entry for symptom diary maintenance. Future research to explore factors influencing app adoption among different groups of patients is warranted.

## Introduction

Chronic heart diseases (CHDs) pose a significant global health challenge, with cases doubling from 271 million in 1990 to 523 million in 2019, leading to a rise in related deaths and disability-adjusted life years, particularly in regions where rates had previously declined [1]. Addressing this global scenario necessitates urgent attention to implementing currently available policies and interventions. However, widespread implementation of interventions that are effective in prevention and management of CHD is hindered by factors such as limited accessibility and affordability, demanding innovative solutions [2,3]. Another barrier to effective management of CHD is the lack of personalized care planning tailored to patient priorities and social contexts, which are vital in providing high quality, patient-centered care [4,5].

Digital health technologies offer promising solutions to overcome some of these barriers [6-8], enhancing clinical outcomes and inducing behavioral changes among individuals with CHD [9-11]. Recent evidence suggests favorable cost-effectiveness outcomes for interventions using digital health technologies, which may aide in the optimization of health care resource usage [12]. Given the necessity for users to take an active role, the widespread adoption and sustained usage of digital health interventions may pose challenges [13,14]. Aligning technology with user preferences increases the probability that intended populations adopt and enjoy its use [15]. Therefore, understanding user preferences is imperative for the successful implementation and sustained use of digital health interventions.

There are numerous methods used to elicit preferences in health preference research. Among these, choice-based methods such as discrete choice experiments (DCEs) are arguably the best known and most commonly used, offering valuable means to systematically analyze user preferences [15,16]. With the rise in the focus toward patient-centered care, DCEs are increasingly used in a range of health policy, planning and resource allocation decisions across disease prevention, diagnosis and treatment, access to services, and health care employment [17,18]. In the framework of a DCE, respondents evaluate alternatives characterized by attribute-level combinations, selecting their preferred option [16]. Choice modeling analysis, rooted in “Random Utility Theory” [19], assumes that this preferred option presents the highest utility for the respondent and can, therefore, quantify preferences and discern overall inclinations toward attribute levels [19].

Our study aimed to investigate the preferences of Australians living with CHD regarding the features of a mobile health app, designed to assist them. We believe that our findings can inform the development of preference-informed mobile apps, enhancing adoption and sustained usage and ultimately improving health outcomes.

## Methods

### Study Design

**Figure 1.**
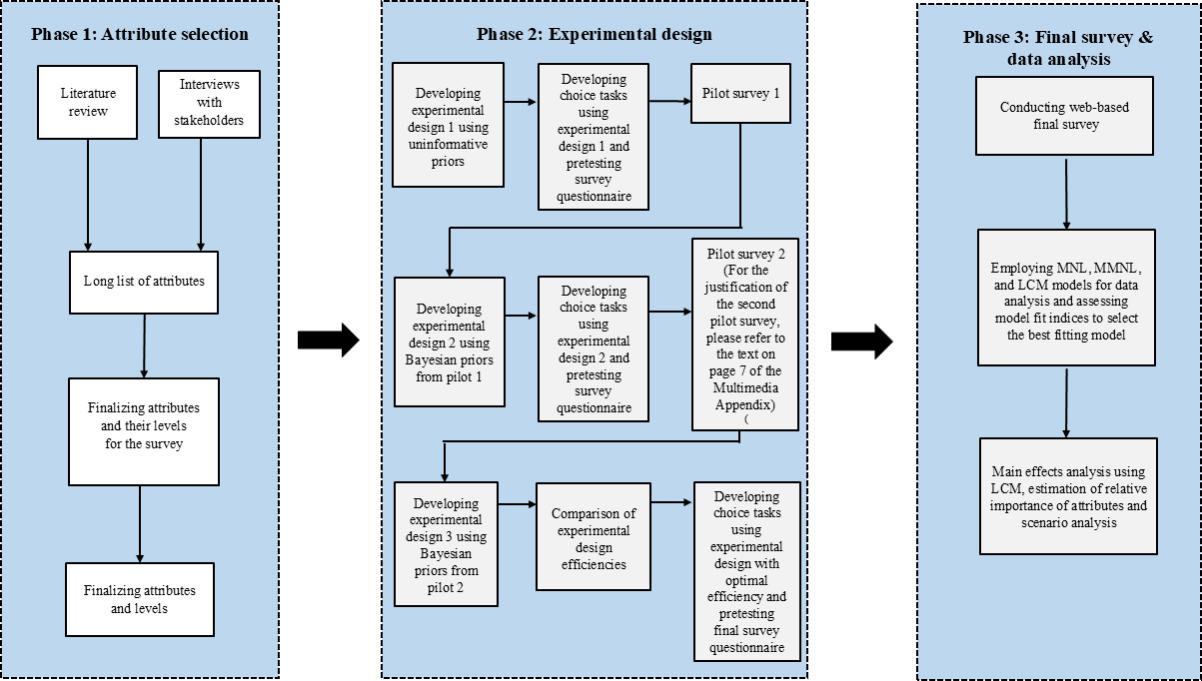
Overview of the study methods. MNL: multinomial logit; MMNL: mixed multinomial logit; LCM: latent class model.

The manuscript was prepared in accordance with the DIRECT (Discrete Choice Experiment Reporting Checklist) checklist for DCEs, as detailed in Table S1 in Multimedia Appendix 1. We conducted the study using three phases: (1) attribute selection, (2) experimental design, and (3) final survey and data analysis, as illustrated in Figure 1.

### Phase 1: Attribute Selection

#### Literature Review to Explore Key Attributes

As an attribute-based experiment, the validity of a DCE heavily relies on appropriately specifying attributes and their levels [17]. However, there is no standard process for identifying and selecting attributes in DCE that may influence the decision of interest [20]. In stage 1, we reviewed existing literature to identify the factors that may affect the use of mobile apps developed for various diseases. The search strategy is shown in Table S2 in Multimedia Appendix 1. The search resulted in 32 papers published between 2012 and 2023, with 23 selected for our final analysis. We identified 38 attributes related to mobile apps from these papers. Figure S1 and Table S3 in Multimedia Appendix 1 provide the PRISMA (Preferred Reporting Items for Systematic Reviews and Meta-Analyses) diagram for the review and the list of attributes identified from the review, respectively.

#### Interviews With Stakeholders

In alignment with previously published recommendations, qualitative work was conducted during attribute development [21]. We interviewed 7 individuals with CHD and 8 health care professionals on the web or via telephone. The discussions yielded themes centered on the user-friendly nature of the app, the capacity of the app to assist in self-monitoring of disease conditions, the need for personalized health education, concerns about data security, and considerations regarding subscription charges. Finally, 2 more attributes were added to the list generated from the stage 1 literature review, bringing the total to 40 attributes.

#### Finalizing the Attributes and Their Levels

Including all attributes identified through literature review and talking to stake holders are impractical due to respondent cognitive burden and sample size limitations [20]. Therefore, authors SK and SH, with backgrounds in health economics and medicine, respectively, reviewed and condensed the list to 7 relevant and suitable attributes by excluding irrelevant ones and merging related ones. A panel of experts including a general cardiologist, a cardiac electrophysiologist, 3 health economists, and an implementation scientist evaluated the proposed 7 attributes and their levels. They determined the significance and applicability of these attributes, offering feedback on the proposed levels through several rounds of evaluation. Four attributes with the highest total scores were selected through group deliberation, each refined with 3 levels based on feedback. Table 1 details these attributes, definitions, levels, and expected preferences.

**Table 1.** Attributes and their levels selected for the study.

Attribute	Working definition	Levels	Expected direction^a^
Training	The level of training required to use the mobile app for the first time.	Easy to use and requires no training.	Reference
		Usable after a basic training for 15 minutes.	−
		Usable after an advanced training for 30 minutes.	−−
Monitoring	Ability of the mobile app to monitor blood pressure and heart rhythm and provide recommendations on action.	The app cannot measure your blood pressure or heart rhythm.	Reference
		The app can measure your blood pressure and heart rhythm but does not provide recommendations on what you should do next.	+
		The app can measure your blood pressure or heart rhythm and provide recommendations on what you should do next.	++
Health education	Availability and nature of health education messages within the mobile app.	Health educational messages are not available in the app.	Reference
		Health educational messages in the app are generalized (not tailored to your individual needs).	+
		Health educational messages in the app are tailored to your individual needs.	++
Symptom diary	Ability of the mobile app to function as a diary to record symptoms or signs by the user.	Keeping a diary of symptoms over time cannot be done in the app.	Reference
		Keeping a diary of symptoms over time can be done, but it is limited to specific questions in the app.	+
		Keeping a diary of symptoms over time can be done in the app without any restrictions.	++

a This column shows the expected direction for participants’ preferences for each attribute level. The “+” and “++” symbols indicate positive direction, while the negative symbols in the cells above indicate negative direction. The number of symbols is an indication of its strength. For example, ++ indicates “strongly positive” while + indicates “positive.”

### Phase 2: Experimental Design

#### Developing Experimental Design 1 Using Uninformative Priors

In the context of DCE, experimental designs pertain to how options, comprising attributes and their respective levels, are presented to participants [22]. While full factorial designs encompass all possible combinations of attribute levels [23], they can be extensive, necessitating large sample sizes or many choice questions per respondent. In studies with a high number of possible choice question combinations, fractional factorial designs, a subset of attribute-level combinations, are recommended [23] and were selected for this study. Our study comprised a 2-alternative design using 4 attributes, each with 3 levels, yielding 81 possible profiles (3^4^) and 3240 combinations of choice questions [3^4^×(3^4^–1)/2] [24]. We chose a D-optimal design over an orthogonal design for its capacity to produce accurate parameter estimates with a smaller sample size [25].

One important consideration in D-optimal design is specifying the number of choice tasks or the design size [26]. According to the formula “number of attribute levels/(number of alternatives-1),” a minimum design size of 6 per block was required [27]. While larger design sizes typically improve statistical efficiency, they can compromise response efficiency [23]. We assessed the normalized D-error for various design sizes beyond the minimum requirement and chose a design size of 16 rows based on the percentage reduction in the normalized D-error (Table S4 in Multimedia Appendix 1). In addition, we used 2 blocks, presenting only 8 choice tasks per respondent, to further enhance response efficiency.

D-optimal designs require specifying parameter priors for each attribute level [28]. While informed priors generally lead to more efficient designs, applying incorrect priors may compromise the expected efficiency compared with uninformative priors [29]. To address this, we generated informative priors from a pilot survey among 67 respondents, using small directional prior values in the prepilot design. The last column of Table 1 indicates the assumed direction for all attribute levels, and small directional prior values used in this exercise are available in Table S5 in Multimedia Appendix 1.

Dummy coding was used to code attribute levels categorically, with the most basic level serving as the reference to interpret results [30]. The position of the opt-out alternative in the choice task was randomly varied so that all 3 alternatives had an equal chance of appearing in different positions. This was done to prevent order effects dependent on position [31].

We used Ngene software (Econometric Software, Inc) to generate our pilot experimental design [32]. The selected design was evaluated for attribute-level balance and minimal overlap, resulting in the experimental design for the first pilot survey. A design is considered balanced when each level of an attribute appears an equal number of times [33], and overlap refers to the repetition of specific attribute levels across a set of alternatives [22]. The Ngene code for our prepilot design is shown in Figure S2 in Multimedia Appendix 1, and an illustration of the Ngene design is shown in Figure S3 in Multimedia Appendix 1.

#### Developing and Pretesting the Survey Questionnaire

**Figure 2.**
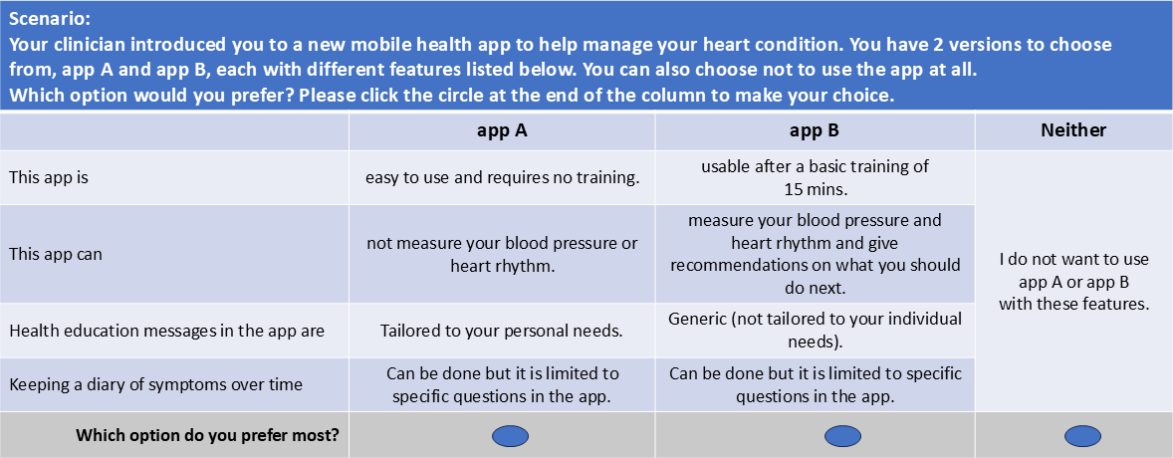
A choice task presented to respondents in this study.

The selected attribute levels were then transformed to ensure meaningful presentation to participants. The survey questionnaire was pretested to ensure clarity and meaningful presentation to participants. Purposeful sampling was used to select nonacademic staff members from the research team’s department for in-person pretesting, enabling direct feedback on paper copies of the experiment. In response to feedback, we refined the wording of specific levels to ensure clear communication of the intended meaning of attribute levels, as illustrated in Figure 2.

Subsequently, a web-based pilot test was conducted among adults aged 18 years and older with CHD, recruited through the PureProfile platform, to estimate Bayesian priors for the final survey design. Each choice task began with an introductory scenario, prompting respondents to envision a mobile app offering to assist them with their CHD. In this unlabeled experiment, the alternatives were referred to as mobile app A and B. Following best practice recommendations in health DCEs, respondents were allowed to opt out if the presented combinations of attribute levels for either app did not align with their preferences [16,34].

While “Random Utility Theory” suggests that respondents opt out only when presented with less attractive alternatives, research indicates that decisions are influenced by motives beyond maximizing personal utility [35-38]. Hence, we adopted a dual response design: if respondents chose not to use any app with the presented features, they were then presented with the same choice task without the “no mobile app” alternative and forced to make a choice. This design mitigates potential power loss and minimizes opt-outs for reasons other than seeking the highest personal utility [34]. The survey design with the forced choice task is depicted in Figure S4 in Multimedia Appendix 1.

#### Pilot Surveys and Selection of the Experimental Design for the Final Survey

The pilot survey aimed to gather informative priors for the subsequent experimental design and to pretest the questionnaire for user-friendliness and clarity. Table S7 in Multimedia Appendix 1 presents the Bayesian priors derived from the pilot survey.

The study focused on adults aged 18 years and older with CHD, as the mobile app was specifically designed for this target population. These criteria also accounted for differences in disease management strategies for younger patients younger than 18 years, making certain features of the app less applicable to them. Participants were recruited through PureProfile [39], an Australian based secure web application frequently used by research academics for web-based surveys that sources its members through diverse online and offline channels. These channels include internal referral programs, paid acquisition, social media, public relations, search engine marketing, offline marketing, and location-based registration [40].

In the absence of specific guidelines for pilot survey sample size estimations, we collected data from 32 participants based on our prior DCE project experience [41]. Pilot survey data were analyzed using the multinomial logit (MNL) model, recognized as the fundamental choice model [23]. We used Nlogit, a widely used commercial software for choice modeling [42], for the analyses. Detailed analysis methods and results are shown in Table S6 and Figure S5 in Multimedia Appendix 1.

### Phase 3: Final Survey and Statistical Analysis

Determining the right sample size relies on factors such as question format, task complexity, result precision, population diversity, participant availability, and the need for subgroup analysis [33,43]. Orme [44] recommended a minimum sample size of 300. Marshall et al [45] observed that the average sample size for health care DCE published from 2005 to 2008 was 259, with nearly 40% ranging between 100 and 300 respondents. Another review studying methods of DCE studies conducted among primary health care professionals found a median sample size of 294 across 34 studies [46]. Accordingly, we opted for a sample size of 300, which also satisfied the minimum sample size estimated using efficiency parameters of the experimental design (highest Sb mean estimate × number of blocks) [25] except for 1 attribute level. Participants were recruited via PureProfile, adhering to eligibility criteria consistent with the pilot test, which included adults aged 18 years and older diagnosed with CHD. Data collection via the web-based survey was completed over a period of 21 days.

#### Main Effects Analysis

Our survey design, prompting respondents who initially selected the “neither” alternative to subsequently choose either app A or B, yielded 2 distinct datasets: unconditional data, capturing free choice, and conditional data, reflecting forced choices. In this study, we considered combining the conditional and unconditional datasets to be inappropriate due to observed differences in participant decision-making processes. This may contradict the principles of random utility theory essential to DCE [47]. Combining conditional and unconditional datasets also raises analytical concerns regarding the potential for biased parameter estimates [48], skewed demand modeling, and inconsistencies in reference data values. Moreover, in scenarios where opting out is a realistic market option and predicting uptake is critical, such as in our study, an unconditional demand model is recommended [47]. It is for these reasons that our main analysis was restricted to the unconditional data. However, results for conditional and combined data are available in Figures S6 and S7 in Multimedia Appendix 1.

In our primary analysis, we used the MNL, mixed multinomial logit model, and latent class model (LCM), assessing model fit using Log Likelihood Ratio and Akaike information criterion per observation to identify the most suitable model. We selected LCM for the main analysis as it had the best model fit indices [27]. Table S8 in Multimedia Appendix 1 presents the outcomes of this comparison. Latent class model assumes that parameter coefficients are distributed among individuals with a discrete distribution, leading to a finite number of classes, each with specific behavioral implications. While respondents are not deterministically assigned to any particular class, they display a probability, known as class assignment probability, of belonging to each class based on sociodemographic characteristics [27]. Subsequently, within each defined latent class, an MNL model was applied.

In the LCM analysis, we explored various model configurations with different numbers of classes (2 and 3) and sociodemographic covariates for class assignments. We initially selected essential sociodemographic covariates for integration into the model through consensus. Subsequently, their retention in the model was determined based on statistical significance [49]. The final model selection was ultimately guided by model fitness indices and logical coherence. The Nlogit code for the chosen LCM analysis is shown in Figure S8 in Multimedia Appendix 1.

The final LCM estimated the importance that participants placed on each attribute level compared with its reference level (part-worth) [30]. To understand the importance of each attribute on the total utility of the mobile app, we estimated relative importance of attributes [50], using parameter coefficients from the base MNL model. This calculation is shown in Table S9 and Figure S9 in Multimedia Appendix 1.

#### Scenario Analysis

Our scenario analysis explored the potential adoption of 3 mobile apps using the base MNL model. The basic app featured the most rudimentary levels for all attributes. The second scenario depicted a mobile app currently used to enhance cardiac rehabilitation in specific private cardiac clinics in Queensland, Australia [51]. This app necessitated basic training, offered facilities for vital sign recording, provided generalized health education, and allowed users to input information into the app as a symptom diary without any restrictions. The third scenario envisioned an advanced app capable of providing the highest levels for each attribute. Analyses were carried out using Nlogit version 6.0 [42], and results are presented as percentage changes from the base share for each scenario. Nlogit code for the scenario analyses is available in Figure S10 in Multimedia Appendix 1.

### Ethical Considerations

This study was approved by the university human research ethics committee of Queensland University of Technology, Australia (reference no. 5732). Respondents were provided with a participant information sheet within the web-based survey, and written consent was obtained prior to engagement with the choice tasks. Access to the survey was restricted to individuals who provided consent. Participants were monetarily compensated for their time in accordance with the terms and conditions of the survey platform, PureProfile [39]. Confidentiality of participants was maintained by anonymizing the data and presenting findings in an aggregated format.

## Results

### Participant Characteristics

Our sample of 302 participants had a mean age of 50.5 (SD 18.2) years, with a slight majority of males (169/302, 56.0%). Most participants (181/302, 59.9%) had CHD for more than 2 years, and 34.1% (103/302) used a mobile app for their condition. While 45.0% (136/302) expressed interest in future app use, 14% (42/302) did not. Geographically, participants were from all Australian states, with the highest representation coming from New South Wales (86/302, 28%) and Victoria (82/302, 27%), the 2 most populous states in Australia [52]. Median survey completion time was 6.8 (IQR 4.6-10.3) minutes. More sociodemographic details are shown in Table 2.

**Table 2.** Sociodemographic and clinical characteristics of participants (N=302).

Characteristics	Number	Frequency (%)
Age (years; mean 50.5, SD 18.2 years)		
18‐24	16	5
25‐34	61	20
35‐44	58	19
45‐54	36	12
55‐64	42	14
65‐74	54	18
>75	35	12
Sex		
Male	169	56
Female	133	44
Level of education		
High school not completed	21	7
High school completed	74	24
Undergraduate	132	44
Postgraduate	75	25
Employment		
Full-time employed	147	49
Part-time employed	53	17
Unemployed	7	2
Disability pension	14	5
Retired	77	25
Other	3	1
Prefer not to say	1	0.3
Annual gross income		
Less than US $12,500 (Aus $20,000)	18	6
US $20,000-US $28,210 (Aus $20,000-Aus $45,000)	71	23
US $28,210-US $37,614 (Aus $45,001-Aus $60,000)	43	14
US $37,614-US $56,421 (Aus $60,001-Aus $90,000)	67	22
US $56,421-US $75,228 (Aus $90,001-Aus $120,000)	44	15
US $75,228-US $94,035 (Aus $120,001-Aus $150,000)	38	13
More than US $94,035 (Aus $150,000)	21	7
State/territory		
Australian Capital Territory	8	3
New South Wales	86	28
Northern Territory	1	0.3
South Australia	22	7
Victoria	82	27
Queensland	65	21
Western Australia	34	11
Tasmania	4	1
Type of heart disease		
Heart rhythm abnormality/pacemaker insertion	60	20
Ischemic heart disease/blocked arteries	71	23
Heart failure/heart weakness	40	13
Cardiomyopathy/heart muscle disease	46	15
Heart valve disease/valve replacement	29	10
Other	56	18
Duration since the diagnosis of heart disease (years)		
<1	46	15
1‐2	75	25
>2	181	60
Previous use of a mobile health app		
I have used a mobile health app before, and I find it useful.	103	34
I have used a mobile health app before, and I did not find it useful.	21	7
I have not used a mobile health app before, but I would like to use one.	136	45
I have not used a mobile health app before, and I do not think I will use one in the near future.	42	14

### Main Effects Analysis

All respondents completed a minimum of 8 choice tasks (unconditional data). Due to the nature of the survey, the total number of choice tasks completed varied among respondents. In total, there were 2803 choice observations from the 302 participants. Most respondents (172/302, 56.9%) answered only 8 choice tasks, indicating that they never selected the “neither” alternative throughout the survey. Conversely, 5.6% (17/302) of participants chose the ’neither’ alternative in all 8 primary choice tasks, resulting in 16 choice tasks for each. The distribution of “neither” alternative for the study sample is shown in Table S10 in Multimedia Appendix 1.

Table 3 presents the outcomes of the LCM, illustrating parameter estimates for the class assignment model and coefficients for each attribute level. Our results identified 2 latent classes within our study sample with 2 distinct preference behavior patterns. Conceptually, LCM operates assuming that preferences are shaped by both observable attributes and unobservable, or latent, heterogeneity [27]. This latent heterogeneity is presumed to represent distinct “preference groups” or “classes” within the sample, with individuals probabilistically assigned to these classes. The model delineated 2 latent classes based on four sociodemographic variables: (1) age, (2) level of education, (3) previous usage of mobile health apps, and (4) perception of the usefulness of mobile health apps. Participants with a higher number of “neither” selections did not exhibit a higher probability of belonging to any specific class identified by LCM analysis (Figure S11 in Multimedia Appendix 1).

Class 1 comprised a higher probability of 85.3% (257/302) for respondents. At the population level, older respondents with education beyond high school, prior experience with mobile health apps, and a positive perception of their utility are more likely to be classified into class 1 than class 2. This probability is predominantly influenced by the individual’s perception of the usefulness of a mobile app (β coefficient 2.9), followed by the level of education (β coefficients 1.2) and previous experience with mobile apps (β coefficients 1.2).

For instance, a 65-year-old individual living with CHD, possessing education beyond high school, prior experience with health apps, and perceiving them as useful, exhibits a 99.7% probability of belonging to class 1. In contrast, a 65-year-old patient with CHD with only high school education, no prior app experience, and a negative perception of app utility has a 60.3% probability of belonging to class 1. Conversely, a 32-year-old patient with CHD with high school education, no prior app experience, and a negative perception of app utility has a 63.9% probability of belonging to class 1. Detailed calculations of class-specific utility and probabilities are shown in Table S11 in Multimedia Appendix 1. On the contrary, membership in class 2 showed no discernible preference for either adopting an app or abstaining from it (app A: −1.18, 95% uncertainty interval [UI] −2.36 to 0.006; app B: −0.78, 95% UI −1.99 to 0.42).

Parameters reported in Table 3 indicate the preferences at the population level, given the 2 classes identified. Respondents in class 1 preferred adopting a mobile app (β coefficient for app A 0.74, 95% UI 0.41-1.06; β coefficient for app B 0.53, 95% UI 0.22-0.85). For them, all attributes contributed positively to the utility of using a mobile app except for the training required before using the app. As anticipated, the preference for advanced training was lower (β coefficient −0.48, 95% UI −0.61 to −0.36) than basic training. Notably, the preference between having no training and undergoing basic training did not reach statistical significance. Respondents also preferred an app capable of providing recommendations on their next steps after monitoring vital signs (β coefficient 1.45, 95% UI 1.26-1.64). Even without recommendations, the ability to monitor blood pressure and heart rhythm retained a significant preference (β coefficient 1.07, 95% UI 0.88-1.26), surpassing the preference for an app that cannot monitor vital signs. These respondents also preferred to receive health education messages tailored to their individual needs (β coefficient 0.50, 95% UI 0.36-0.64), followed by receiving generalized health education messages (β coefficient 0.29, 95% UI 0.13-0.44) compared with not receiving health education messages at all. The ability to use the mobile app as a symptom diary without any restrictions (β coefficient 0.58, 95% UI 0.41-0.76) was preferred compared with limiting it to app-generated specific prompts (β coefficient 0.23, 95% UI 0.06-0.41) or not being able to use the app as a symptom diary. In contrast, individuals in class 2 exhibited no particular inclination toward any of the presented attribute levels.

**Table 3.** Results of the selected latent class model (log-likelihood function = −2050.644, Akaike information criterion/N=1.718).

	Class 1		Class 2	
	β Coefficient (95% UI^a^)	SE	β Coefficient (95% UI)	SE
Class properties				
Class membership	85.3%	—^b^	14.7%	—
Constant	−1.53 (−3.36 to 0.31)	0.94	Reference	—
Age (years)	0.03^c^ (0.002 to 0.06)	0.01	—	—
Education above high school	1.23^c^ (0.27 to 2.19)	0.49	—	—
Having used a mobile health app before	1.16^c^ (0.12 to 2.21)	0.53	—	—
Positive perception of usefulness of mobile health apps	2.92^d^ (1.88 to 3.95)	0.53	—	—
Alternative specific constant				
Neither	Reference	—	Reference	—
Mobile app A	0.74^d^ (0.41 to 1.06)	0.16	−1.18^e^ (−2.36 to 0.006)	0.60
Mobile app B	0.53^d^ (0.22 to 0.85)	0.16	−0.78 (−1.99 to 0.42)	0.61
Training				
No training	Reference	—	—	—
Basic training	0.008 (−0.13 to 0.14)	0.07	0.30 (−0.27 to 0.88)	0.29
Advanced training	−0.49^d^ (−0.61 to −0.36)	0.06	−0.06 (−0.99 to 0.86)	0.47
Monitoring of vital signs				
No Monitoring	Reference	—	—	—
Monitor without recommendations	1.07^d^ (0.88 to 1.26)	0.09	−0.08 (−1.15 to 0.98)	0.54
Monitor with recommendations	1.45^d^ (1.26 to 1.64)	0.10	−0.91 (−2.00 to 0.18)	0.55
Health education				
No health education	Reference	—	—	—
Generalized health education	0.29^d^ (0.14 to 0.44)	0.08	−0.27 (−1.16 to 0.61)	0.45
Individualized health education	0.50^d^ (0.36 to 0.64)	0.07	−0.57 (−1.37 to 0.23)	0.41
Maintaining a symptom diary				
Not possible	Reference	—	—	—
Possible but restricted to app questions	0.23^d^ (0.06 to 0.41)	0.09	−0.30 (−1.10 to 0.50)	0.41
Possible without any restrictions	0.58^d^ (0.41 to 0.76)	0.09	−0.28 (−1.21 to 0.66)	0.48

a UI: uncertainty interval.

b Not applicable.

c Significance at 5% level.

d Significance at 1% level.

e Significance at 10% level.

To identify observable heterogeneity across different types of heart diseases, a subgroup analysis was conducted for each disease type. The analysis revealed that participants with most heart disease types, except heart failure and cardiomyopathy, generally disliked advanced training. In contrast, individuals with heart failure did not show a preference for facilities to monitor vital signs. Preferences for other attributes varied across disease types, as detailed in Table S12 in Multimedia Appendix 1. However, these results should be interpreted with caution, as the study was not powered for post hoc subgroup analyses.

### Relative Importance of Attributes

**Figure 3.**
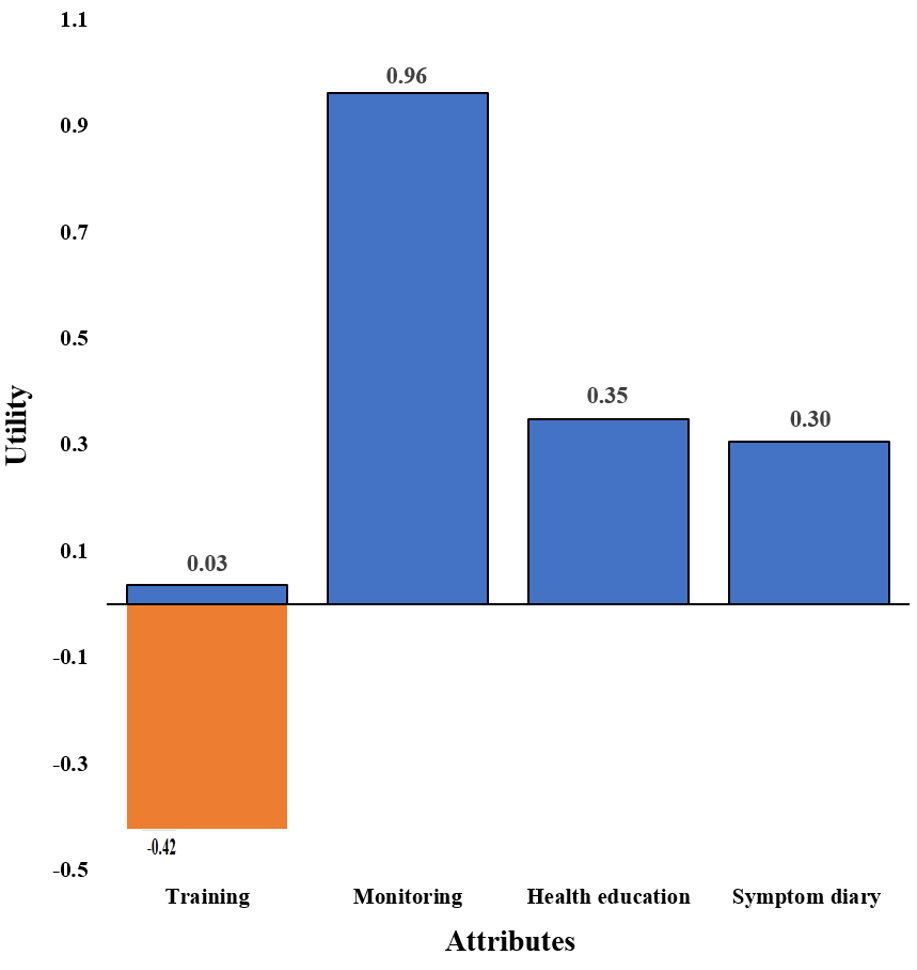
Utility ranges for attributes.

As shown in Figure 3, utility ranges were positive for all attributes except for training.

We estimated what app features were most valued by the survey participants by calculating the relative importance of attributes. Accordingly, the most influential feature affecting participants’ decision to adopt a mobile app was its ability to monitor vital signs (relative importance of 46.4%). Participants next weighed the level of training required to navigate the app (relative importance of 22.1%). The delivery method of health education (relative importance of 16.8%) and app’s ability to function as a symptom diary (relative importance of 14.7%) were considered less in their decision-making process. However, it is essential to note that the magnitude of relative importance is not directional. A higher relative importance does not necessarily indicate that respondents preferred it, but merely that they considered that attribute important. Calculation of relative importance of attributes is detailed in Table S9 in Multimedia Appendix 1.

### Scenario Analysis

We assessed the uptake of 3 versions of a mobile health app via scenario analyses (Table 4). The probability of participants adopting an app with basic features (scenario 1) was 84%. Upon upgrading the app features to those delineated in scenario 2, the adoption rate increased by 8.1%. However, with further enhancement of attributes to create an advanced app (scenario 3), the adoption rate increased only by 7.9%, which is a marginal drop compared with scenario 2. This slight decline could be due to the requirement for advanced training in the app in scenario 3. Training was estimated to hold the second-highest relative importance among all attributes, with advanced training being unfavorably viewed by respondents in class 1, who comprised a higher probability (85%) of the sample.

**Table 4.** Results of the scenario analysis.

	Scenario 1(basic app)	Scenario 2(resembles a current app in use)^a^	Scenario 3(advanced app)
Training	No training required	Basic training required	Advanced training required
Monitoring	No monitoring of vital signs	Monitors vital signs but provides no recommendations	Monitors and provides recommendations
Health education	No health education	Generalized health education	Personalized health education
Maintaining a symptom diary	Cannot enter information	Can enter restricted information	Can enter information without any restrictions
Base share (%)	84	84	84
Scenario share (%)	84	92.1	91.9
Change of share from base to scenario (%)	N/A^b^	+8.1	+7.9

a A mobile app currently used to enhance cardiac rehabilitation in a specific private cardiac clinic in Queensland, Australia [51].

b N/A: not applicable.

## Discussion

### Principal Findings

Our LCM analysis uncovered 2 distinct latent groups among our survey respondents. Class 1 members, who are typically well-educated older adults with prior experience in app usage and a positive perception of app utility, expressed a preference for apps that are easy to navigate with minimal or no training. In addition, they favored features such as vital sign monitoring, feedback provision, personalized health education, and symptom diary functionality. On the other hand, class 2 members did not show a clear preference for adopting or rejecting mobile apps based on the attributes outlined in our survey; their preferences for attribute levels were indifferent. Among the presented app features, the ability to monitor vital signs and provide feedback primarily influenced the decision to adopt an app.

### Comparison With Previous Work

As this was the first study investigating a population of individuals living with cardiac diseases, we were unable to compare our findings with studies from similar cohorts of patients. Our study found an encouraging 84% potential adoption rate for a mobile health app assisting individuals with CHD, even with basic features. Preferences for adopting mobile apps to assist with self-management have been demonstrated in various other health-related contexts, such as depression and anxiety [53], interventions for alcohol [54], diabetes mellitus [55], and smoking cessation [56].

The app feature of monitoring blood pressure and heart rhythm garnered strong preference among the majority, particularly when it provided recommendations. This attribute also exhibited the highest relative importance, indicating the significance individuals living with CHD attribute to monitoring their vital signs. Similar preferences for feedback and suggestions in mobile health apps have been reported in research on other chronic conditions such as HIV [57], cancer [58], and metabolic syndrome [59].

Most participants expressed indifference toward basic training versus no training, but they opposed mobile apps requiring advanced training, indicating a preference for apps that are user-friendly and straightforward to navigate. This finding was further confirmed by participants assigning significant importance to the level of training required, ranking it second highest in relative importance. Recent evidence on mobile health interventions for chronic diseases has underscored simplicity and ease of navigation as crucial factors in determining the effective use of the intervention [11,60]. In addition, ease of use has been recognized as a significant influence on the decision to purchase mobile health apps [61]. Majority of respondents also favored personalized health education over general information, aligning with previous studies emphasizing user preferences for personalization of app functionalities [56,59,62-65].

It is widely recognized that user characteristics have a great influence on sustained use of technologies designed for behavior change [66,67]. In addition to preference toward app features, our findings shed light on the influence of user characteristics on technology adoption. Class 1 membership, characterized by well-educated older adults with prior app experience and a positive perception of app utility, demonstrated a strong inclination toward adopting mobile apps for CHD management. A recent longitudinal study identified 4 dimensions that could influence the adoption and sustained use of mobile health apps, including the “user’s assessment of mobile health apps” [60]. Our findings on class assignment demographics support this conclusion, indicating that individuals with previous mobile app experience and positive perceptions of app utility were more inclined to adopt the app. The identification of older adults with class 1, rather than younger individuals, was an unexpected finding, as young adults are generally presumed to be more receptive to and adopt digital health interventions [68,69].

Individuals more likely to be classified into class 2—often younger, with a high school education or below, lacking familiarity with mobile apps, and perceiving them as not useful—showed no clear preference for either adopting or abstaining from using an app based on the attributes presented in our survey. For this demographic, the features outlined in our study may not be pertinent or adequate to stimulate app adoption. Future research investigating populations with similar characteristics would be beneficial in elucidating the reasons for mobile app nonadoption, which may encompass specific barriers such as unfamiliarity with mobile technology or skepticism regarding its utility.

### Limitations

While we aimed to enhance internal validity, our study has inherent limitations. It focused on 302 Australian patients with CHD, relying on self-reported data in a web-based survey. While our sample encompassed a diverse range of ages, types of CHD, and nearly equal representation of both males and females, our findings may not generalize well to dissimilar populations, including other diseases groups, cultural contexts or people with dissimilar digital literacy, or rates of smartphone ownership. Further research on varied populations could enrich the understanding of mobile health app acceptance and feature preferences.

Selecting attributes and specifying attribute levels are inherently subjective and may not comprehensively capture the spectrum of factors influencing individuals’ preferences for mobile health apps. Despite our efforts to enhance this process by reviewing literature and consulting stakeholders for contextual insights, it is possible that certain attributes or levels important to patients may have been overlooked or insufficiently represented in our study design. Nevertheless, we believe that the attributes outlined in our study could still exert a substantial influence on real-world app adoption and usage. We recommend ensuring patient representation within the expert panel in future research endeavors.

Although we pretested the survey questionnaire to enhance understandability, biases linked to respondents’ interpretation of choice tasks may persist, influencing their choice preferences. In addition, in practice, individuals may consider a broader array of factors and trade-offs, such as technological literacy, access to health care services, and socioeconomic status, when adopting mobile apps. In addition, some participants may not have considered all presented attributes in their decision-making process (attribute nonattendance). This may result in biased coefficient estimates and a skewed understanding of respondent preferences, an inherent bias in DCE [70,71].

### Conclusions

The majority of respondents expressed a preference for adopting an app, even with basic features. Adoption rates were further boosted when app attributes included easy navigation, vital sign monitoring, feedback provision, personalized health education, and flexible data entry for symptom diary maintenance. However, it seems that these adoption rates may vary based on population demographics, with a minority showing reluctance to adopt apps with the features outlined in our study. Future research, encompassing both quantitative and qualitative approaches to explore the factors influencing app adoption among the demographics identified in our study, which are less receptive to mobile apps, is likely to contribute significantly to advancing this field.

Given that the majority of individuals living with CHD are inclined to adopt mobile health apps to manage their condition, we are optimistic that our findings will provide valuable insights in designing preference-informed mobile health apps. This, in turn, has the capacity to enhance adoption rates and promote sustained engagement with mobile apps among individuals living with CHD, thereby potentially contributing to improvements in clinical outcomes.

## Supplementary material

10.2196/58556 Multimedia Appendix 1 Search strategy and PRISMA (Preferred Reporting Items for Systematic Reviews and Meta-Analyses) flowchart for the review; long list of attributes; directional priors for the prepilot test; Ngene and Nlogit codes for prepilot, pilot, and final surveys; model efficiency parameters; relative importance of attribute calculations; and Nlogit code for scenario analysis and class-specific profiling calculations.

## Abbreviations

CHD: chronic heart disease
DCE: discrete choice experiment
DIRECT: Discrete Choice Experiment Reporting Checklist
LCM: latent class model
MNL: multinomial logit
PRISMA: Preferred Reporting Items for Systematic Reviews and Meta-Analyses
UI: uncertainty interval
