# Supplementary material for: Preferences for Mobile App Features to Support People Living With Chronic Heart Diseases: Discrete Choice Study
Source: JMIR Mhealth Uhealth. 2025 Apr 25;13:e58556. doi: 10.2196/58556 (PMC12047850; doi:10.2196/58556)
Supplement: Multimedia Appendix 1 [file mhealth-v13-e58556-s001.pdf]

Supplementary file

Preferences for mobile app features to support people living with chronic heart diseases: A discrete choice study.

Sumudu Hewage [1] , Sameera Senanayake [1,2,3] , David Brain [1], Michelle Allen [1] , Steven McPhail [1,4], William Parsonage [1,5], Tomos Walters [6,7], Sanjeewa Kularatna [1,2,3]

[1] Australian Centre for Health Services Innovation and Centre for Healthcare Transformation, School of Public Health and Social Work, Queensland University of Technology, Australia.

[2] Health services and systems research, Duke-NUS Medical School, Singapore.

[3] National Heart Research Institute Singapore, National Heart Centre, Singapore

[4] Digital Health and Informatics Directorate, Metro South Health, Queensland, Australia

[5] Cardiology department, Royal Brisbane and Women's Hospital, Queensland, Australia

[6] Queensland Cardiovascular group, Queensland, Australia

[7] Faculty of Medicine, University of Queensland, Queensland, Australia

Table s1. DIRECT checklist reporting guidelines

Item		Section/ subsection
Purpose and rationale		
1	Describe the real-world context and decision-maker that the hypothetical choice context seeks to replicate or inform	Introduction
2	Provide a rationale for using a DCE to answer the research question	Introduction
Attributes and levels		
3	Describe how attributes and levels were derived (e.g. literature review, interviews, focus groups, expert input)	Methods<br>Phase 1: Attribute selection
4	Provide the final list of attributes and levels	Table 1
Experimental design		
5	Report the number of alternatives per choice set and whether they were labelled or unlabelled	Developing and pre-testing the survey questionnaire
6	Describe response options (e.g. forced choice, opt-out, status quo)	Developing and pre-testing the survey questionnaire
7	Describe the type of experimental design (e.g. orthogonal, D-efficient, Bayesian efficient, partial profile)	Phase 2: Experimental design
8	Describe which effects are identified in the design (e.g. main effects, higher order interactions, functional form)	Main effects analysis
9	Describe the number of choice sets, blocks and choice sets per block	Developing Experimental design 1 using uninformative priors
10	Indicate how the experimental design was obtained (software, catalogue, other)	Phase 2: Experimental design
Survey design		
11	Provide a sample choice set and the instructions and background information given to respondents (e.g. providing the survey as an appendix)	Figure 2,<br>Developing and pre-testing the survey questionnaire
12	Report any randomisation (e.g. choice set order, attribute order, alternative order, framing effects)	Developing Experimental design 1 using uninformative priors
13	Describe what was checked in piloting (e.g. understanding, respondent burden, timing, wording)	Developing and pre-testing the survey questionnaire, Pilot surveys and selection of the experimental design for the final survey
14	Report whether information from the pilot was used to update the experimental design (e.g. priors, functional form of attributes) or survey design	Pilot surveys and selection of the experimental design for the final survey
Sample and data collection		

Item		Section/ subsection
15	Report respondent inclusion/exclusion criteria	Phase 3. Final survey and statistical analysis
16	Describe how data were collected (e.g. mail, personal interview, web survey)	Phase 3. Final survey and statistical analysis
17	Report the response rate or cooperation rate, if possible	Response rate not possible to estimate.
18	Report the final sample size and how the sample size was determined	Phase 3. Final survey and statistical analysis
19	Describe respondent characteristics and representativeness of target population, if known	Results: Participant characteristics
Econometric analysis		
20	Indicate coding of data (e.g. effects, dummy, continuous) including definitions	Phase 2: Experimental design. Developing Experimental design 1 using uninformative priors
21	Report whether any respondents were removed and why (e.g. suspected fraudulent responses, rationality tests)	Main effects analysis
22	Provide the rationale for model choice (e.g. conditional logit, mixed logit, latent class) and assumptions (e.g. error variance)	Main effects analysis
23	Report model specification	Main effects analysis
Reporting of results		
24	Report the model performance, goodness of fit (if comparing models)	Main effects analysis, table s8 in the supplementary file
25	Describe methods used for analysis of model results (e.g. calculation of marginal rate of substitution, attribute relative importance, welfare gain)	Main effects analysis, attribute relative importance, scenario analysis
26	Report measures of precision for the output(s) of interest (e.g. confidence intervals) and how these were derived	Results: Main effects analysis

Table s2: Search strategy for the review

Domain	Search terms	Strategy
Mobile health	Mobile phone*[Title/Abstract] OR Smart phone*[Title/Abstract] OR Cell phone[Title/Abstract] OR Cellphone[Title/Abstract] OR Cell-phone[Title/Abstract] OR e-health[Title/Abstract] OR eHealth[Title/Abstract] OR mHealth[Title/Abstract] OR m-Health[Title/Abstract] OR mobile health[Title/Abstract] OR Digital health[Title/Abstract] OR Digital technolog*[Title/Abstract]	#1
	AND	
Applications	App*[Title/Abstract] OR Application*[Title/Abstract]	#2
	AND	
App features	feature*[Title/Abstract] OR characteristic*[Title/Abstract] OR option*[Title/Abstract] OR Attribute*[Title/Abstract] OR propert*[Title/Abstract] OR trait*[Title/Abstract] OR element*[Title/Abstract]	#3
	AND	
DCE	Discrete choice experiment[Title/Abstract] OR Discrete-choice experiment[Title/Abstract] OR Conjoint analysis[Title/Abstract] OR patient* preference*[Title/Abstract] OR user Preference*[Title/Abstract]	#4
	NOT	
	support tool[Title/Abstract]) OR decision tool[Title/Abstract] OR guide*[Title/Abstract] OR protocol*"[Title/Abstract] OR pathway[Title/Abstract] OR recommendation*"[Title/Abstract]	#5
Final strategy	#1 AND #2 AND #3 AND #4 NOT #5	

Figure s1: PRISMA diagram for the review.

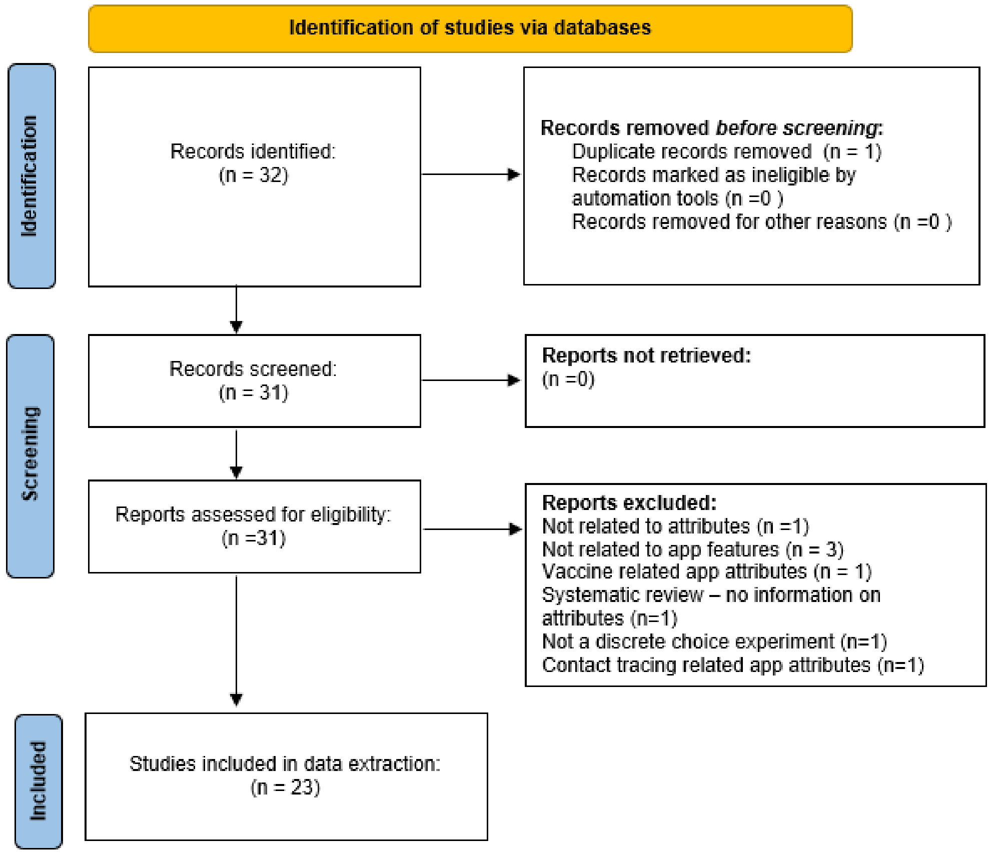

From: Page MJ, McKenzie JE, Bossuyt PM, Boutron I, Hoffmann TC, Mulrow CD, et al. The PRISMA 2020 statement: an updated guideline for reporting systematic reviews. BMJ 2021;372:n71. doi: 10.1136/bmj.n71

Table s3: The list of attributes identified from the literature review (items 1 to 47) and focus group discussions (1 to 49).

	**Attributes**
1	Instructions are simple and clear/ ease of use/ easy navigation
2	Ability to record information/ symptoms/ periodic self-measurements
3	Trustworthiness of information provided by the app
4	Ability to monitor disease related signs
5	Health education messages – availability, way of presentation
6	Cost – subscription fee and payment options
7	Type of service platform (operable on any platform)
8	System failure /frequency of breakdown
9	Ability to communicate with care provider
10	Service tailoring/ personalised services
11	Frequency of new information added
12	Data safety/ security
13	Possibility of data exchange between caring health professionals
14	Proven effectiveness of the App

15	Peer support platform/ Opportunities for contact with other users
16	Recommended by whom (treating healthcare professional/ marketing team)
17	Mode of content delivery (letters/ graphics/ coloured/ black and white/ voice messages)
18	Availability of information of other services<br>(e.g.: smoking cessation clinics, nearby AAA clubs, links to health authority websites)
19	Who can review data/ data sharing policy
20	Ability to download own personal data
21	Mobile data consumption
22	Phone storage consumption
23	Ability to self-screen
24	Ability to integrate with hospital medical records
25	Ability to detect disease complications
26	Length of modules/ number of questions the user must fill in
27	Mode of data entry: drop down menus, manual entry, voice recording, drag and drop
28	Origin of the app
29	Ability to control who can view data
30	Dashboard appearance
31	Attractiveness of the home page
32	Self-report assessment
33	Booking and video-visit systems
34	Ability to create own well-being plan/ goals
35	Supervision of care giver involvement
36	Motivation to sustained use
37	Ability to track progress
38	Reinforcement based on user feedback
39	Work-out specific features tailored for the disability
40	Profile personalisation options
41	Video demonstrations of exercises
42	Push notifications
43	Ability to schedule appointments with the clinicians
44	Availability of additional resources
45	Gamification features that allowed for competition with self and/or others
46	Attitudes of healthcare professionals towards the app
47	Approval stamp by the health department/ authority
48	Provide more recent updates about the disease management
49	Pop-up advertisements

Table s4: Normalised D-errors for different design sizes.

Design size (number of rows)	D-error after 24 hours	Normalised D-error	D-error difference from the previous design
8	0.394262	3.154096	[redacted]
10	0.314623	3.14623	0.007866
12	0.262603	3.151236	-0.005006
16	0.19588	3.13408	0.017156
30	0.104094	3.12282	0.01126

Table s5: Small directional prior values used in the pre-pilot Ngene design.

Attribute	Level	Directional priors
Training	Easy to use and requires no training	Ref
	Usable after a basic training for 15 minutes	-0.001
	Usable after an advanced training for 30 minutes	-0.002
Symptom monitoring	The App cannot measure your blood pressure and heart rhythm	Ref
	The App can measure your blood pressure and heart rhythm but does not provide recommendations for your action	0.001
	The App can measure your blood pressure and heart rhythm and provides recommendations for your action	0.002
Health education messages	are not available in the App	Ref
	are generalised (not tailored to your needs)	0.001
	are tailored to your individual needs	0.002
Keeping a diary of symptom	cannot be done in the App	Ref
	can be done, but is limited to specific questions in the App	0.001
	Can be done and is not limited to specific questions in the App	0.002

Figure s2: Ngene code for the pre-pilot design with uninformative small directional priors.

```
Design
   ;alts = appA, appB, neither
   ;rows = 12
   ;block = 3
   ;eff = (mnl,d)
   ;alg = mfederov
   ;model :
   U(appA) =
           + b_training.effect      [-0.001|-0.002] * TRAINING      [3,2,1]
           + b_monitoring.effect    [0.001|0.002]   * MONITORING    [3,2,1]
           + b_HE.effect            [0.001|0.002]   * HE            [3,2,1]
           + b_typing.effect        [0.001|0.002]   * TYPING        [3,2,1]
           + b_cost                 [-0.001]        * COST          [0,10,30,50]/
   U(appB) =
           + b_training.effect      * TRAINING      [3,2,1]
           + b_monitoring.effect    * MONITORING    [3,2,1]
           + b_HE.effect            * HE            [3,2,1]
           + b_typing.effect        * TYPING        [3,2,1]
           + b_cost                 * COST          [0,10,30,50]
   $
```

Figure s3: An illustration of the experimental design used for the first pilot survey.

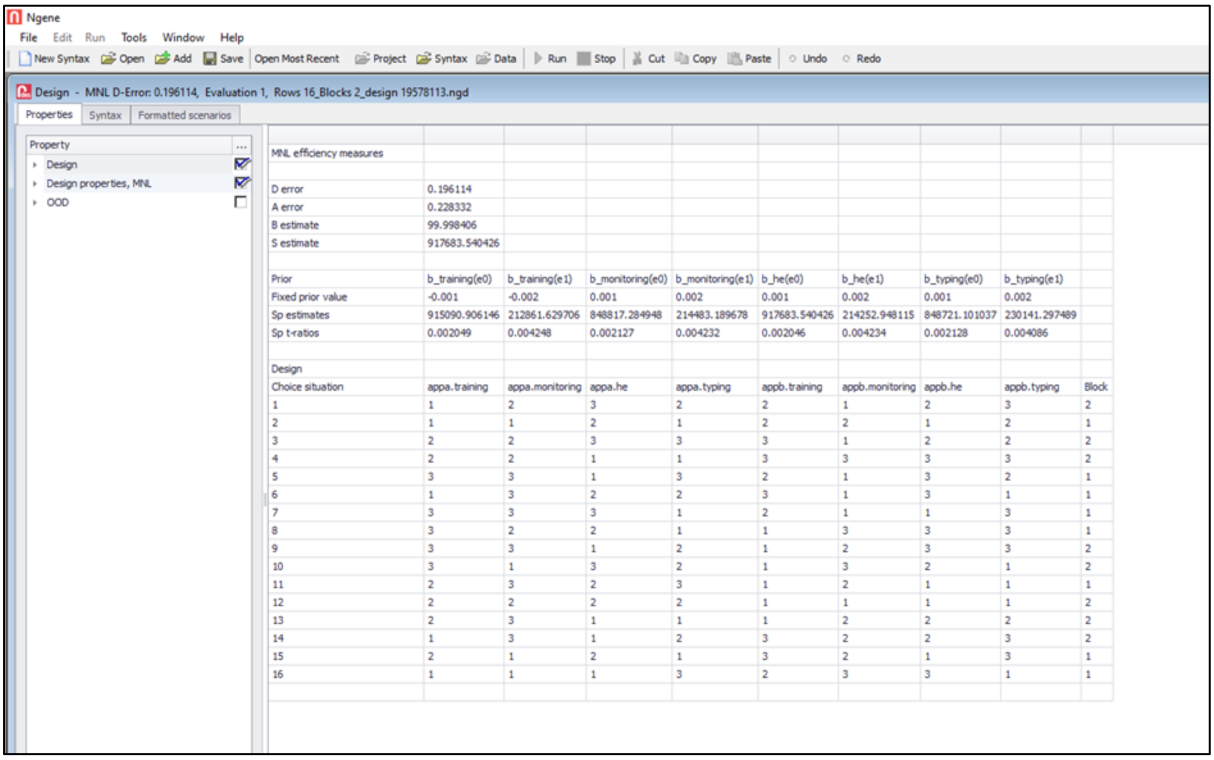

MNL efficiency measures									
D error	0.196114								
A error	0.228332								
B estimate	99.998406								
S estimate	917683.540426								
Prior	b_training(e0)	b_training(e1)	b_monitoring(e0)	b_monitoring(e1)	b_he(e0)	b_he(e1)	b_typing(e0)	b_typing(e1)	
Fixed prior value	-0.001	-0.002	0.001	0.002	0.001	0.002	0.001	0.002	
Sp estimates	915090.906146	212861.629706	848817.284948	214483.189678	917683.540426	214252.948115	848721.101037	230141.297489	
Sp t-ratios	0.002049	0.004248	0.002127	0.004232	0.002046	0.004234	0.002128	0.004086	
Design									
Choice situation	appa.training	appa.monitoring	appa.he	appa.typing	appb.training	appb.monitoring	appb.he	appb.typing	Block
1	1	2	3	2	2	1	2	3	2
2	1	1	2	1	2	2	1	2	1
3	2	2	3	3	3	1	2	2	2
4	2	2	1	1	3	3	3	3	2
5	3	3	1	3	2	1	3	2	1
6	1	3	2	2	3	1	3	1	1
7	3	3	3	1	2	1	1	3	1
8	3	2	2	1	1	3	3	3	1
9	3	3	1	2	1	2	3	3	2
10	3	1	3	2	1	3	2	1	2
11	2	3	2	3	1	2	1	1	1
12	2	2	2	2	1	1	1	1	2
13	2	3	1	1	1	2	2	2	2
14	1	3	1	2	3	2	2	3	2
15	2	1	2	1	3	2	1	3	1
16	1	1	1	3	2	3	3	1	1

Figure s4: Survey design with the forced choice tasks.

Imagine a new mobile App is introduced to you to help you with the follow-up care for your heart condition. There are 2 types of mobile Apps, app A and app B, for you to choose from. Alternatively, you can choose NOT to use a mobile app. The table below present both Apps with their characteristics. Which option would you prefer?

	**Mobile App A**	**Mobile App B**	**No mobile app**
The App is	easy to use and requires no training	usable after a basic training of 15 minutes	I do not want to use App A or App B with those features.
The App can	not measure your blood pressure or heart rhythm	measure your blood pressure and heart rhythm, and give recommendations on what you should do next	
Health education messages	in the App are tailored to your individual needs	in the App are generic (not tailored to your specific needs)	
Keeping a diary of symptoms over time	can be done but it is limited to specific questions in the App.	can be done and it is limited to specific questions in the App.	
**Which option do you prefer the most?**			

Display the following question underneath the table ONLY AFTER the participant has selected an option in the choice task shown above.

In a hypothetical scenario where you MUST choose a mobile App, which App would you choose from the options presented to you before?

	**Mobile App A**	**Mobile App B**
The App is	easy to use and requires no training	usable after a basic training of 15 minutes
The App can	not measure your blood pressure or heart rhythm	measure your blood pressure and heart rhythm, and give recommendations on what you should do next
Health education messages	in the App are tailored to your individual needs	in the App are generic (not tailored to your specific needs)
Keeping a diary of symptoms over time	can be done but it is limited to specific questions in the App.	can be done and it is limited to specific questions in the App.
**Which option do you prefer the most?**		

Ngene code for design comparison

We selected an experimental design with 16 rows (figure s3) for the first pilot survey. The results of this pilot survey yielded a Sb mean estimate of 4760 for the level 2 of 'Health Education' attribute, representing the minimum number of respondents required to attain a statistically significant estimate for this parameter using Bayesian priors with one block [1]. This suggested a considerable level of uncertainty surrounding the result for level 2 of the Health Education attribute unless the required larger sample size was achieved in the final survey.

As this sample size was not feasible in the final survey, we conducted a second pilot test involving 34 respondents to explore if the efficiency measures for the experimental design, including sb mean estimate, would improve. The resulting experimental design revealed an Sb mean estimate exceeding 7200 for level 2 of the attribute "Typing" (indicating the ability to enter data to maintain a symptom diary), presenting a similar issue to that encountered with the previous experimental design. Consequently, we amalgamated datasets from both Pilot 1 and Pilot 2 surveys and the same process was replicated. Despite the enhancement in MNL efficiency measures observed with the combined dataset, the Sb mean estimate still surpassed 1000 for one attribute-level.

Given the unfeasible sample size, we relied on efficiency parameters of experimental designs to determine the optimal choice among designs derived from both pilot surveys and the combined dataset. This entailed assessing the D-error of various experimental designs while applying the same Bayesian priors across all designs [2]. Subsequently, we selected the experimental design derived from the combined dataset, which exhibited the lowest D-error of 0.25 (table s5). Ngene code utilised for design comparison is presented in figure s5.

Table s6: Efficiency comparison of experimental designs

	Fixed	Mean	Std dev.	Median	Minimum	Maximum
D1 design with D1 priors	0.196114	[redacted]				
D1 design with D2 priors	0.374793	0.386066	0.03812	0.385072	0.279079	0.502925
D2 design with D2 priors	0.251437	0.271795	[redacted]			
D2 design with D3 priors	0.271338	0.323699	0.050708	0.313915	0.22294	0.51738
D3 design with D3 priors	0.223626	0.280493	[redacted]			
D3 design with D4 priors	0.346978	0.352249	0.028404	0.351986	0.271543	0.424668
D4 design with D4 priors	0.241551	0.252761	[redacted]			
D1 design with D4 priors	0.365402	0.37115	0.036039	0.370526	0.291685	0.47848
D2 design with D4 priors	0.245221	0.256145	0.015492	0.255166	0.221315	0.305671
D3 design with D4 priors	0.346978	0.352249	0.028404	0.351986	0.271543	0.424668
D4 design with D4 priors	0.241551	0.252761	[redacted]			

Figure s5: Ngene code utilised for design comparison presented in table s5.

```
Design
;alts = appA, appB, neither
;rows = 16
;block = 2
;eff = (mnl,d,mean)
;eval = Eval_1.xlsx
;model :
U(appA) =
b_training.effect [(n,-0.53152,0.22209)|(n,-0.82365,0.23732)] * TRAINING [3,2,1]
+ b_monitoring.effect [(n,0.38913,0.49741)|(n,-0.00841,0.46258)] * MONITORING [3,2,1]
+ b_HE.effect [(n,0.01499,0.23337)|(n,-0.49564,.22903)] * HE [3,2,1]
+ b_typing.effect [(n,0.34457,0.33512)|(n,-0.18257,0.31939)] * TYPING[3,2,1]
/
U(appB) =
b_training.effect * TRAINING [3,2,1]
+ b_monitoring.effect * MONITORING [3,2,1]
+ b_HE.effect * HE [3,2,1]
+ b_typing.effect * TYPING [3,2,1]
/
U(neither) = ASC_n [(n,-1.70901,0.80793)]
$
```

Table s7. Bayesian priors derived from the pilot survey and utilised in the post-pilot experimental design.

Attribute	Level	Co-efficient	Standard error
Training	Easy to use and requires no training	reference	
	Usable after a basic training for 15 minutes	-0.07237,	0.13749
	Usable after an advanced training for 30 minutes	-0.12399,	0.14349
Monitoring	The App cannot measure your blood pressure or heart rhythm	reference	
	The App can measure your blood pressure and heart rhythm but does not provide recommendations for your action	0.74842	0.17066
	The App can measure your blood pressure and heart rhythm and provides recommendations for your action	1.08799	0.17622
Health education	are not available in the App	reference	
	are generalised (not tailored to your needs)	-0.05556	0.13787
	are tailored to your individual needs	0.28265	0.13826
Keeping a diary of symptoms	cannot be done in the App	reference	
	can be done, but is limited to specific questions in the App	0.31658	0.15224
	Can be done and is not limited to specific questions in the App	0.71844	0.17066

Figure s6. Results for the LCM analysis with 2 Classes for the conditional dataset

```
-----------------------------------------------------------------------------
Latent Class Logit Model
Dependent variable                 CHOICE
Log likelihood function        -223.14767
Restricted log likelihood      -268.24796
Chi squared [ 26](P= .000)       90.20059
Significance level                 .00000
McFadden Pseudo R-squared        .1681291
Estimation based on N =     387, K =   26
Inf.Cr.AIC  =     498.3 AIC/N =     1.288
---------------------------------------
               Log likelihood R-sqrd R2Adj
No coefficients  -268.2480  .1681 .1082
Constants only can be computed directly
               Use NLOGIT ;...;RHS=ONE$
At start values  -253.7932  .1207 .0574
Note: R-sqrd = 1 - logL/Logl(constants)
Root Likelihood:Geom. Mean of P^  .5618
---------------------------------------
Response data are given as ind. choices
Number of latent classes =            2
Average Class Probabilities
     .188  .812
LCM model with panel has      130 groups
Variable number of obs./group =PAN
Number of obs.=   387, skipped    0 obs
--------+--------------------------------------------------------------------
        |                  Standard            Prob.      95% Confidence
  CHOICE|  Coefficient       Error       z    |z|>Z*         Interval
--------+--------------------------------------------------------------------
        |Random utility parameters in latent class -->>  1...............
 ASC_C|1|     9.11715**      4.11058     2.22  .0266     1.06057  17.17374
   TR2|1|   -61.9248       .9973D+11      .00 1.0000 ***********  ***********
   TR3|1|   -6.14939**       2.51687    -2.44  .0146   -11.08237  -1.21642
   TY2|1|    2.32207         2.67567      .87  .3855    -2.92215   7.56628
   TY3|1|    3.85909**       1.91101     2.02  .0434      .11359   7.60460
    M2|1|   -4.74476**       2.08576    -2.27  .0229    -8.83278   -.65674
    M3|1|   -3.25971         2.58230    -1.26  .2068    -8.32091   1.80150
   HE2|1|   -9.78498***      3.68673    -2.65  .0080   -17.01084  -2.55913
   HE3|1|   -4.07650         2.60951    -1.56  .1182    -9.19104   1.03804
        |Random utility parameters in latent class -->>  2...............
 ASC_C|2|     .55153          .58427      .94  .3452     -.59363   1.69668
   TR2|2|     .20407          .49787      .41  .6819     -.77174   1.17987
   TR3|2|    -.50823          .52083     -.98  .3292    -1.52903    .51257
   TY2|2|   -1.18724***       .40762    -2.91  .0036    -1.98617   -.38832
   TY3|2|    -.99091**        .39808    -2.49  .0128    -1.77113   -.21069
    M2|2|     .68331**        .34025     2.01  .0446      .01643   1.35020
    M3|2|     .63227*         .37121     1.70  .0885     -.09529   1.35983
   HE2|2|     .62221          .43511     1.43  .1527     -.23060   1.47501
   HE3|2|     .30083          .36617      .82  .4113     -.41685   1.01851
        |This is THETA(01) in class probability model....................
  _ONE|1|   -21.2361        24.81300     -.86  .3921    -69.8686   27.3965
  _AGE|1|     .19612          .22861      .86  .3910     -.25194    .64418
 _SEX1|1|    1.30628         1.58092      .83  .4086    -1.79227   4.40482
 _EDU1|1|    2.54308         2.92272      .87  .3842    -3.18535   8.27151
 _EMP1|1|    6.23746         8.42096      .74  .4589   -10.26731  22.74224
_DSDUR|1|    5.22818         5.56374      .94  .3474    -5.67656  16.13292
_APPUS|1|   -32.6865       .1255D+07      .00 1.0000 ***********  ***********
_USFUL|1|    1.79438         2.90234      .62  .5364    -3.89410   7.48286
        |This is THETA(02) in class probability model....................
  _ONE|2|        0.0     .....(Fixed Parameter).....
  _AGE|2|        0.0     .....(Fixed Parameter).....
 _SEX1|2|        0.0     .....(Fixed Parameter).....
 _EDU1|2|        0.0     .....(Fixed Parameter).....
 _EMP1|2|        0.0     .....(Fixed Parameter).....
_DSDUR|2|        0.0     .....(Fixed Parameter).....
_APPUS|2|        0.0     .....(Fixed Parameter).....
_USFUL|2|        0.0     .....(Fixed Parameter).....
--------+--------------------------------------------------------------------
nnnnn.D-xx or D+xx => multiply by 10 to -xx or +xx.
***, **, * ==>  Significance at 1%, 5%, 10% level.
Fixed parameter ... is constrained to equal the value or
had a nonpositive st.error because of an earlier problem.
Model was estimated on Feb 15, 2024 at 00:41:38 PM
-----------------------------------------------------------------------------
```

Figure s7. Results for the LCM analysis with 2 Classes for the combined dataset

```
-----------------------------------------------------------------------------
Latent Class Logit Model
Dependent variable CHOICE
Log likelihood function -2399.17591
Restricted log likelihood -4511.25447
Chi squared [ 27 d.f.] 4224.15712
Significance level .00000
McFadden Pseudo R-squared .4681799
Estimation based on N = 2803, K = 27
Inf.Cr.AIC = 4852.4 AIC/N = 1.731
Model estimated: May 29, 2024, 14:12:59
Constants only must be computed directly
 Use NLOGIT ;...;RHS=ONE$
At start values -2650.0370 .0947******
Response data are given as ind. choices
Number of latent classes = 2
Average Class Probabilities
 .472 .528
LCM model with panel has 302 groups
Variable number of obs./group =PAN2
Number of obs.= 2803, skipped 0 obs
--------+--------------------------------------------------------------------
 | Standard Prob. 95% Confidence
 CHOICE| Coefficient Error z |z|>Z* Interval
--------+--------------------------------------------------------------------
 |Utility parameters in latent class -->> 1
ASC_A|1| .70351*** .24393 2.88 .0039 .22542 1.18160
 TR2|1| .03834 .07570 .51 .6125 -.11003 .18671
 TR3|1| -.45627*** .07965 -5.73 .0000 -.61239 -.30016
 TY2|1| .47226*** .12344 3.83 .0001 .23033 .71420
 TY3|1| .88861*** .13413 6.62 .0000 .62571 1.15150
 M2|1| 1.52117*** .15935 9.55 .0000 1.20885 1.83348
 M3|1| 2.00652*** .18045 11.12 .0000 1.65283 2.36020
 HE2|1| .33145*** .07495 4.42 .0000 .18456 .47835
 HE3|1| .65084*** .08541 7.62 .0000 .48345 .81824
ASC_B|1| .49572** .24586 2.02 .0438 .01384 .97759
ASC_C|1| .08365 .28282 .30 .7674 -.47066 .63796
 |Utility parameters in latent class -->> 2
ASC_A|2| -.16553 .25222 -.66 .5116 -.65987 .32880
 TR2|2| -.11543 .11546 -1.00 .3174 -.34172 .11086
 TR3|2| -.68659*** .12960 -5.30 .0000 -.94061 -.43257
 TY2|2| -.24102 .14812 -1.63 .1037 -.53133 .04928
 TY3|2| -.16998 .15433 -1.10 .2707 -.47246 .13249
 M2|2| -.05793 .16353 -.35 .7232 -.37844 .26259
 M3|2| -.28550 .18659 -1.53 .1260 -.65121 .08021
 HE2|2| -.06837 .11835 -.58 .5635 -.30033 .16359
 HE3|2| -.15197 .12901 -1.18 .2388 -.40483 .10090
ASC_B|2| -.19388 .25305 -.77 .4436 -.68985 .30208
ASC_C|2| .38469*** .13191 2.92 .0035 .12615 .64322
 |This is THETA(01) in class probability model.
Constant| -33.2763 .1189D+08 .00 1.0000 *********** ***********
_AGE1|1| -3.44961 .6743D+08 .00 1.0000 *********** ***********
_EDU1|1| -4.95139 .8115D+08 .00 1.0000 *********** ***********
_APPUS|1| 2.12423 .1790D+09 .00 1.0000 *********** ***********
_USFUL|1| 1.19104 .8424D+08 .00 1.0000 *********** ***********
 |This is THETA(02) in class probability model.
Constant| 0.0 .....(Fixed Parameter).....
_AGE1|2| 0.0 .....(Fixed Parameter).....
_EDU1|2| 0.0 .....(Fixed Parameter).....
_APPUS|2| 0.0 .....(Fixed Parameter).....
_USFUL|2| 0.0 .....(Fixed Parameter).....
```

--------+--
Note: nnnnn.D-xx or D+xx => multiply by 10 to -xx or +xx.
Note: ***, **, * ==> Significance at 1%, 5%, 10% level.
Fixed parameter ... is constrained to equal the value or
had a nonpositive st.error because of an earlier problem.

Table s8. AIC/N comparison

Model fit indices	MNL analysis	MMNL analysis	LCM analysis (without Class assignment)	
			2 classes	3 classes [a]
AIC/N	1.982	1.809	1.72	1.678
Log Likelihood ratio	-2384.27771	-2166.86045	-2049.87896	-1981.54159
Average Class probability			Class 1: 85.4%<br>Class 2: 14.6%	Class 1: 73.8%<br>Class 2: 18.6%<br>Class 3: 0.76%

[a] Although LCM analysis with 3 classes has the lowest model fit indices, we considered LCM analysis with 2 classes based on sensible class probabilities and covariate significance.

Figure s8: Nlogit code for the final LCM analysis with 2 Classes and four covariates (age,edu1,AppUse1,Usful)

```
Nlogit
;lhs=choice,cset,alt
;choices= appA, appB, neither
;lcm= age, edu1, AppUse1, Usful
;pts=2
;pds=Pan1
;checkdata
;model:
 U(appA) = ASC_A + TR2*tr2 + TR3*tr3 + TY2*ty2 + TY3*ty3 + M2*m2 + M3*m3 + HE2*he2 + HE3*he3/
 U(appB) = ASC_B + TR2*tr2 + TR3*tr3 + TY2*ty2 + TY3*ty3 + M2*m2 + M3*m3 + HE2*he2 + HE3*he3
$
```

Figure s9. MNL model output used for the estimation of relative importance of attributes.

```
|-> Nlogit

  ;lhs=choice,cset,alt
  ;choices= appA, appB, neither

  ;lcm= age,edu2,Appuse1,Usful
  ;pts=2

  ;pds=Pan1
  ;checkdata

  ;model:
  U(appA) = ASC_A + TR2*tr2 + TR3*tr3 + TY2*ty2 + TY3*ty3 + M2*m2 + M3*m3 + HE2*he2 + HE3*he3/
  U(appB) = ASC_B + TR2*tr2 + TR3*tr3 + TY2*ty2 + TY3*ty3 + M2*m2 + M3*m3 + HE2*he2 + HE3*he3$
+--------------------------------------------------------+
| Inspecting the data set before estimation.             |
| These errors mark observations which will be skipped.  |
| Row Individual = 1st row then group number of data block |
+--------------------------------------------------------+
No bad observations were found in the sample

Iterative procedure has converged
Normal exit:   4 iterations. Status=0, F=    .2384278D+04

-----------------------------------------------------------------------------
Discrete choice (multinomial logit) model
Dependent variable              Choice
Log likelihood function     -2384.27771
Estimation based on N =   2416, K =  10
Inf.Cr.AIC  =   4788.6 AIC/N =    1.982
---------------------------------------
          Log likelihood R-sqrd R2Adj
ASCs  only  model must be fit separately
            Use NLOGIT ;...;RHS=ONE$
Note: R-sqrd = 1 - logL/Logl(constants)
Root Likelihood:Geom. Mean of P^  .3727
---------------------------------------
Chi-squared[ 8](LR test) =    169.64260
Prob [ chi squared > value ] =    .00000
Response data are given as ind. choices
Number of obs.=  2416, skipped    0 obs
--------+--------------------------------------------------------------------
        |               Standard            Prob.      95% Confidence
  CHOICE|  Coefficient       Error       z    |z|>Z*         Interval
--------+--------------------------------------------------------------------
 ASC_A|1|      .20243        .14919    1.36  .1748    -.08998    .49485
   TR2|1|      .03508        .06440     .54  .5859    -.09113    .16130
   TR3|1|    -.42265***      .06735   -6.28  .0000    -.55466   -.29064
   TY2|1|      .00728        .08478     .09  .9316    -.15889    .17345
   TY3|1|      .30491***     .08828    3.45  .0006     .13188    .47794
    M2|1|      .68379***     .09884    6.92  .0000     .49006    .87751
    M3|1|      .96025***     .10674    9.00  .0000     .75105   1.16945
   HE2|1|      .21192***     .06464    3.28  .0010     .08522    .33862
   HE3|1|      .34771***     .06812    5.10  .0000     .21419    .48123
 ASC_B|1|      .04516        .14839     .30  .7608    -.24567    .33600
--------+--------------------------------------------------------------------
***, **, * ==>  Significance at 1%, 5%, 10% level.
Model was estimated on Feb 19, 2024 at 01:11:49 PM
-----------------------------------------------------------------------------
```

Table s9. Estimation of relative importance of attributes.

Attribute	Highest utility value (co-efficient)	Lowest utility value (co-efficient)	Range	Individual % from total utility range	Percentage importance
Training	0.03508	-0.42265	0.45773	22.10615281	22.11%
Monitoring	0.96025	0	0.96025	46.37544673	46.38%
Health education	0.34771	0	0.34771	16.79271709	16.79%
Symptom diary	0.30491	0	0.30491	14.72568338	14.73%
		Total utility range	2.0706		

Figure s10: Nlogit code for scenario analysis.

Scenario 1:

```
|-> Nlogit
    ;lhs = choice, cset, alt
    ;choices = appA, appB, neither
    ;simulate
    ;scenario: tr1(appA, appB)=1 / ty1(appA, appB)=1 / m1(appA, appB)=1 / he1(appA, appB)=1
    ;model:
    U(appA) = ASC_A + TR2*tr2 + TR3*tr3 + TY2*ty2 + TY3*ty3 + M2*m2 + M3*m3 + HE2*he2 + HE3*he3 /
    U(appB) = ASC_B + TR2*tr2 + TR3*tr3 + TY2*ty2 + TY3*ty3 + M2*m2 + M3*m3 + HE2*he2 + HE3*he3
    $
```

Scenario 2:

```
|-> Nlogit
    ;lhs = choice, cset, alt
    ;choices = appA, appB, neither
    ;simulate
    ;scenario: tr2(appA, appB)=1 / ty3(appA, appB)=1 / m2(appA, appB)=1 / he2(appA, appB)=1
    ;model:
    U(appA) = ASC_A + TR2*tr2 + TR3*tr3 + TY2*ty2 + TY3*ty3 + M2*m2 + M3*m3 + HE2*he2 + HE3*he3 /
    U(appB) = ASC_B + TR2*tr2 + TR3*tr3 + TY2*ty2 + TY3*ty3 + M2*m2 + M3*m3 + HE2*he2 + HE3*he3
    $
```

Scenario 3:

```
|-> Nlogit
    ;lhs = choice, cset, alt
    ;choices = appA, appB, neither
    ;simulate
    ;scenario: tr3(appA, appB)=1 / ty3(appA, appB)=1 / m3(appA, appB)=1 / he3(appA, appB)=1
    ;model:
    U(appA) = ASC_A + TR2*tr2 + TR3*tr3 + TY2*ty2 + TY3*ty3 + M2*m2 + M3*m3 + HE2*he2 + HE3*he3 /
    U(appB) = ASC_B + TR2*tr2 + TR3*tr3 + TY2*ty2 + TY3*ty3 + M2*m2 + M3*m3 + HE2*he2 + HE3*he3
    $
```

Table s10. Distribution of the ‘neither’ selection by participants out of 8 choice tasks

Number of ‘neither’ choice tasks	Number of participants (n)	Percentage out of total number of participants (n=302)
Selected the ‘neither’ option in 0 out of 8 choice tasks	172	56.9%
Selected the ‘neither’ option in 1 out of 8 choice tasks	49	16.2%
Selected the ‘neither’ option in 2 out of 8 choice tasks	32	10.6%
Selected the ‘neither’ option in 3 out of 8 choice tasks	14	4.6%
Selected the ‘neither’ option in 4 out of 8 choice tasks	6	2.0%
Selected the ‘neither’ option in 5 out of 8 choice tasks	5	1.6%
Selected the ‘neither’ option in 6 out of 8 choice tasks	2	0.6%
Selected the ‘neither’ option in 7 out of 8 choice tasks	5	1.6%
Selected the ‘neither’ option in 8 out of 8 choice tasks	17	5.6%
Selected at least one ‘neither’ option out of 8 tasks	130	43.0%
Total number of ‘neither’ options selected out of 2416 (8*302) choice	387	16.0%

Table s11. Detailed calculations of class-specific utility and probabilities (profiling).

Profile	Utility	Class assignment probability
	U (C1) = constant+(β*Age)+(β*edu)+(β*appUse)+(β*usefulness)<br>U(C2) = 0	P(C1) = Exp C1 utility / Exp C1 utility + Exp C2 utility
a 65-year-old individual with higher education, with previous app use and thinks a mobile app could be useful	U (C1) = - 1.53 + (0.03*65) + (1.23*1) + (1.16*1) + (2.92*1)<br>= 5.73<br>U (C2) = 0	P(C1) = $e^{5.73}$ / $e^{5.73}$ + e^{0}<br>= 307.969268 / 307.969268 + 1<br>= 0.996763432<br>P(C2) = e^{0} / $e^{5.73}$ + e^{0} = 0.004
a 65-year-old individual with education up to high school, who has used mobile health apps previously and thinks a mobile app could be useful:	U (C1) = - 1.53 + (0.03*65) + (1.23*0) + (1.16*1) + (2.92*1)<br>= 45<br>U (C2) = 0	P(C1) = $e^{4.5}$ / $e^{4.5}$ + e^{0}<br>= 90.0171313 / 90.0171313 + 1<br>= 0.989013057<br>P(C2) = e^{0} / $e^{4.5}$ + e^{0} = 0.010986943
a 65-year-old individual with education up to high school, with no previous app use and thinks a mobile app could be useful:	U (C1) = - 1.53 + (0.03*65) + (1.23*0) + (1.16*0) + (2.92*1)<br>= 3.34<br>U (C2) = 0	P(C1) = $e^{3.34}$ / $e^{3.34}$ + e^{0}<br>= 28.2191267 / 28.2191267 + 1<br>= 0.96577<br>P(C2) = e^{0} / $e^{3.34}$ + e^{0} = 0.034
a 65-year-old individual with education up to high school, with no previous app use and thinks a mobile app would not be useful	U (C1) = - 1.53 + (0.03*65) + (1.23*0) + (1.16*0) + (2.92*0)<br>= 0.42<br>U (C2) = 0	P(C1) = $e^{0.42}$ / $e^{0.42}$ + e^{0}<br>= 1.52196156/ 1.52196156 + 1<br>= 0.603483251<br>P(C2) = e^{0} / $e^{0.42}$ + e^{0} = 0.396516749
32-year-old individual with education above high school, with previous app use and thinks a mobile app would be useful:	U (C1) = - 1.53 + (0.03*32) + (1.23*1) + (1.16*1) + (2.92*1)<br>= 4.74<br>U (C2) = 0	P(C1) = $e^{4.74}$ / $e^{4.74}$ + e^{0}<br>= 114.434202 / 114.434202 + 1<br>= 0.99<br>P(C2) = e^{0} / $e^{4.74}$ + e^{0} = 0.01
a 32-year-old individual with education up to high school, with no previous app use and thinks a mobile app would not be useful	U (C1) = - 1.53 + (0.03*32) + (1.23*0) + (1.16*0) + (2.92*0)<br>= -0.57<br>U (C2) = 0	P(C1) = $e^{-0.57}$ / $e^{-0.57}$ + e^{0}<br>= 0.565525439 / 0.565525439 + 1<br>= 0.361236825<br>P(C2) = e^{0} / $e^{-0.57}$ + e^{0} = 0.638763175

Figure s11. Results for the LCM analysis with 'neither' option as a covariate

```
|-> Nlogit
 ;lhs=choice,cset,alt
 ;choices= appA, appB, neither
 ;lcm= age1, edu1, AppUse1, Usful, NTHR
 ;pts=2
 ;pds=Pan1
 ;CheckData
 ;model:
 U(appA) = ASC_A + TR2*tr2 + TR3*tr3 + TY2*ty2 + TY3*ty3 + M2*m2 + M3*m3 + HE2*he2 + HE3*he3/
 U(appB) = ASC_B + TR2*tr2 + TR3*tr3 + TY2*ty2 + TY3*ty3 + M2*m2 + M3*m3 + HE2*he2 + HE3*he3
 $
+-------------------------------------------------------+
| Inspecting the data set before estimation. |
| These errors mark observations which will be skipped. |
| Row Individual = 1st row then group number of data block |
+-------------------------------------------------------+
No bad observations were found in the sample
Normal exit: 4 iterations. Status=0, F= 2384.278

-----------------------------------------------------------------------------
Discrete choice (multinomial logit) model
Dependent variable Choice
Log likelihood function -2384.27771
Estimation based on N = 2416, K = 10
Inf.Cr.AIC = 4788.6 AIC/N = 1.982
Model estimated: May 29, 2024, 17:20:15
R2=1-LogL/LogL* Log-L fncn R-sqrd R2Adj
Constants only must be computed directly
 Use NLOGIT ;...;RHS=ONE$
Chi-squared[ 8] = 169.64260
Prob [ chi squared > value ] = .00000
Response data are given as ind. choices
Number of obs.= 2416, skipped 0 obs
--------+--------------------------------------------------------------------
 | Standard Prob. 95% Confidence
 CHOICE| Coefficient Error z |z|>Z* Interval
--------+--------------------------------------------------------------------
ASC_A|1| .20243 .14919 1.36 .1748 -.08998 .49485
 TR2|1| .03508 .06440 .54 .5859 -.09113 .16130
 TR3|1| -.42265*** .06735 -6.28 .0000 -.55466 -.29064
 TY2|1| .00728 .08478 .09 .9316 -.15889 .17345
 TY3|1| .30491*** .08828 3.45 .0006 .13188 .47794
 M2|1| .68379*** .09884 6.92 .0000 .49006 .87751
 M3|1| .96025*** .10674 9.00 .0000 .75105 1.16945
 HE2|1| .21192*** .06464 3.28 .0010 .08522 .33862
 HE3|1| .34771*** .06812 5.10 .0000 .21419 .48123
ASC_B|1| .04516 .14839 .30 .7608 -.24567 .33600
--------+--------------------------------------------------------------------
Note: ***, **, * ==> Significance at 1%, 5%, 10% level.
-----------------------------------------------------------------------------
Line search at iteration 67 does not improve fn. Exiting optimization.
-----------------------------------------------------------------------------
Latent Class Logit Model
Dependent variable CHOICE
Log likelihood function -1965.60390
Restricted log likelihood -2654.24729
Chi squared [ 26 d.f.] 1377.28678
Significance level .00000
```

McFadden Pseudo R-squared .2594496
Estimation based on N = 2416, K = 26
Inf.Cr.AIC = 3983.2 AIC/N = 1.649
Model estimated: May 29, 2024, 17:20:17
Constants only must be computed directly
Use NLOGIT ;...;RHS=ONE$
At start values -2384.2325 .1756******

Response data are given as ind. choices
Number of latent classes = 2
Average Class Probabilities
.786 .214
LCM model with panel has 302 groups
Variable number of obs./group =PAN1
BHHH estimator used for asymp. variance
Number of obs.= 2416, skipped 0 obs
--------+--
| Standard Prob. 95% Confidence
CHOICE| Coefficient Error z |z|>Z* Interval
--------+--
|Utility parameters in latent class -->> 1
ASC_A|1| .83927*** .18426 4.55 .0000 .47813 1.20041
TR2|1| -.00081 .07398 -.01 .9913 -.14581 .14419
TR3|1| -.50169*** .06836 -7.34 .0000 -.63566 -.36771
TY2|1| .36505*** .09377 3.89 .0001 .18126 .54884
TY3|1| .73835*** .09445 7.82 .0000 .55323 .92348
M2|1| 1.27463*** .10073 12.65 .0000 1.07720 1.47206
M3|1| 1.71663*** .10563 16.25 .0000 1.50960 1.92365
HE2|1| .32763*** .08079 4.06 .0001 .16929 .48596
HE3|1| .58843*** .07733 7.61 .0000 .43687 .74000
ASC_B|1| .63008*** .17952 3.51 .0004 .27823 .98192
|Utility parameters in latent class -->> 2
ASC_A|2| -.69490* .36685 -1.89 .0582 -1.41391 .02412
TR2|2| .16052 .19318 .83 .4060 -.21810 .53914
TR3|2| -.32371 .21075 -1.54 .1245 -.73677 .08935
TY2|2| -.34468 .21906 -1.57 .1156 -.77403 .08466
TY3|2| -.20560 .32252 -.64 .5238 -.83773 .42653
M2|2| .19828 .30197 .66 .5114 -.39357 .79013
M3|2| -.24296 .27512 -.88 .3772 -.78219 .29626
HE2|2| -.12251 .25326 -.48 .6286 -.61890 .37387
HE3|2| -.28692 .21455 -1.34 .1811 -.70743 .13360
ASC_B|2| -.65660* .36657 -1.79 .0733 -1.37508 .06187
|This is THETA(01) in class probability model.
Constant| 596.546 .1061D+09 .00 1.0000 *********** ***********
_AGE1|1| -145.444 .4745D+08 .00 1.0000 *********** ***********
_EDU1|1| 37.7707 .4449D+17 .00 1.0000 *********** ***********
_APPUS|1| -115.066 .2380D+14 .00 1.0000 *********** ***********
_USFUL|1| .37702 2.22448 .17 .8654 -3.98289 4.73693
_NTHR|1| -225.457 .4745D+08 .00 1.0000 *********** ***********
|This is THETA(02) in class probability model.
Constant| 0.0 .....(Fixed Parameter).....
_AGE1|2| 0.0 .....(Fixed Parameter).....
_EDU1|2| 0.0 .....(Fixed Parameter).....
_APPUS|2| 0.0 .....(Fixed Parameter).....
_USFUL|2| 0.0 .....(Fixed Parameter).....
_NTHR|2| 0.0 .....(Fixed Parameter).....
--------+--
Note: nnnnn.D-xx or D+xx => multiply by 10 to -xx or +xx.

--------+--

Table s12. Summary of results for the parameter coefficients across six subgroups by the type of heart disease.

Parameter	Parameter co-efficient (95% uncertainty interval)					
	Heart rhythm disease (n=60)	Ischaemic heart disease (n=71)	Heart failure (n=40)	Cardiomyopathy (n=46)	Valvular heart disease (n=29)	Other heart diseases (n=56)
Alternative specific constant (ASC)						
Neither	Reference					
For mobile app A	0.28 (-0.01,0.58)	-0.16 (- 0.77, 0.44)	0.29 (-0.52,1.10)	0.21 (-0.55,0.97)	-0.07 (-1.08,0.93)	0.71 (-0.05,1.47)
For mobile app B	0.06 (-0.21,0.34)	-0.22 (-0.81,0.37)	-0.02 (-0.82,0.78)	-0.09 (0.87,0.68)	-0.15 (-1.17,0.87)	0.68 (-0.08,1.43)
Training requirements for the app						
No training	Reference					
Basic training	0.25 [a] (0.08,0.40)	0.04 (- 0.22,0.30)	0.19 (-0.16,0.55)	0.02 (-0.30,0.35)	-0.38 (-0.79,0.03)	0.06 (-0.24,0.37)
Advanced training	-0.36 [a] (-0.52,-0.19)	-0.44 [a] (- 0.72,- 0.16)	-0.11 (-0.48, 0.24)	-0.29 (-0.62,0.04)	-0.95 [a] (-1.40,-0.51)	-0.41 [b] (-0.73,-0.09)
Maintaining a symptom diary						
Not possible	Reference					
Restricted to app drop down menu	-0.41 [a] (-0.59, -0.23)	0.10 (- 0.24,0.44)	-0.05 (-0.52,0.41)	0.36 (-0.07,0.79)	0.39 (-0.18,0.96)	-0.52 [b] (-0.94,-0.10)
Possible without any restrictions	-0.04 (-0.21,0.14)	0.42 [b] (0.06,0.78)	0.28 (-0.19,0.75)	0.65 [a] (0.20,1.10)	0.49 (-0.11,1.11)	-0.21 (-0.65,0.23)
Monitoring of vital signs						
No monitoring	Reference					
Monitoring without recommendations	0.54 [a] (0.34,0.73)	0.90 [a] (0.49,1.30)	0.14 (-0.36,0.64)	0.98 [a] (0.45,1.49)	1.18 [a] (0.48,2.00)	0.52 [b] (0.01,1.04)
Monitoring with recommendations	0.85 [a] (0.64, 1.05)	1.35 [a] (0.91,1.78)	0.25 (-0.29,0.80)	1.16 [a] (0.61,1.72)	1.24 [a] (-0.10,0.73)	0.89 [a] (0.34,1.45)
Health education						
No health education	Reference					
Generalised health education	0.27 [a] (0.11, 0.42)	0.31[b] (0.04,0.57)	0.40 [b] (0.04, 0.76)	0.03 (-0.28,0.35)	0.31 (-0.10,0.73)	0.02 (-0.27,0.33)
Personalised health education	0.38 [a] (0.20,0.55)	0.69[a] (0.41,0.97)	0.10 (-0.28,0.48)	0.23 (-0.11,0.57)	0.55 [b] (-1.17,0.87)	0.24 (-0.08,0.57)

[a] significance at 10% [b] significance at 5%

References

[1] J. M. Rose and M. C. J. Bliemer, "Sample size requirements for stated choice experiments," *Transportation,* vol. 40, no. 5, pp. 1021-1041, 2013/09/01 2013, doi: 10.1007/s11116-013-9451-z.

[2] ChoiceMetrics Pty Ltd, *Ngene 1.2 USER MANUAL & REFERENCE GUIDE*. 2018.
